# Impact of air pollution on lung function in cystic fibrosis over a decade in London: a UK CF Registry study

**DOI:** 10.1136/thorax-2024-222710

**Published:** 2026-02-05

**Authors:** Muhammad Saleem Khan, Benjamin Barratt, Bethan Davies, Nicholas J Simmonds, Frédéric B Piel

**Affiliations:** 1 UK Small Area Health Statistics Unit, School of Public Health, Faculty of Medicine, Imperial College London, London, UK; 2 National Institute for Health & Care Research (NIHR) Health Protection Research Unit (HPRU) in Environmental Exposures and Health, Imperial College London, London, UK; 3 Medical Research Council (MRC) Centre for Environment and Health, School of Public Health, Faculty of Medicine, NIHR, London, UK; 4 Environmental Research Group, School of Public Health, Imperial College London, London, UK; 5 Adult Cystic Fibrosis Centre, Royal Brompton Hospital, London, UK; 6 National Heart and Lung Institute, Imperial College London, London, UK

**Keywords:** Cystic Fibrosis

## Abstract

**Objective:**

Despite extensive research on the detrimental effects of air pollution on respiratory diseases like asthma and chronic obstructive pulmonary disease, the impact on people with cystic fibrosis (CF) remains understudied. Our study aimed to quantify the association between air pollution exposure and lung function decline in people with CF in London, UK.

**Methods:**

Using 10 years (2008–2017) of UK Cystic Fibrosis Registry data, we conducted a longitudinal cohort study to evaluate the association between air pollution and the rate of decline in percent predicted forced expiratory volume in 1 second (ppFEV_1_) among people with CF. Residential postcode exposure was based on high-resolution models of particulate matter with a diameter of less than 2.5 μm (PM_2.5_) and nitrogen dioxide (NO_2_) from the London Air Pollution Toolkit. We estimated the temporal decline in ppFEV_1_ in high, medium and low exposure subgroups based on air pollutant concentration tertiles using linear mixed models with random intercepts.

**Results:**

We used 3333 ppFEV_1_ measurements of 393 people with CF (122 children, 271 adults). Over 40% of these people with CF lived in postcodes falling into the most deprived national quintile. For PM_2.5_, the adjusted mean ppFEV_1_ declined by 13.3% (95% CI −24.1% to −4.0%) over the study period in the high-exposure tertile compared with 8.5% (95% CI −11.7% to −6.4%) in the low-exposure tertile. Differences between the exposure groups were less consistent for NO_2_. Children and people with CF with severe genotypes seemed particularly vulnerable.

**Conclusions:**

This study provides novel evidence of the detrimental impact of air pollution on lung function in people with CF. Our findings highlight the importance of addressing air pollution as a modifiable risk factor to improve long-term outcomes of people with CF, and the need for national studies of the impact of environmental factors on CF in the UK.

**WHAT IS ALREADY KNOWN ON THIS TOPIC**

- Non-genetic factors, including environmental exposures, are important modifying contributors to disease severity and progression in cystic fibrosis (CF).
- Few studies have investigated the impact of air pollution on CF, and their findings are inconsistent.

**WHAT THIS STUDY ADDS**

- By using high-quality health data and high-resolution residential modelled exposure over a 10-year period for people with CF living in London, our study addresses key methodological limitations of previous studies.
- Our findings suggest that long-term exposure to particulate matter (PM) may substantially affect lung function in all people with CF.

**HOW THIS STUDY MIGHT AFFECT RESEARCH, PRACTICE OR POLICY**

- Non-pharmacological approaches to improving CF outcomes remain important for all people with CF, regardless of their eligibility to CF protein ‘modulator’ therapies.
- Our findings confirm that a better understanding of the impact of environmental factors on all people with CF could help prevent complications.
- Given the wide range of sources of PM, including indoors, it is important to identify which pollutants are the most harmful for all people with CF.
- Further policy measures targeting sources of PM are needed to reduce inequalities in exposures and health risks.

## Introduction

 Cystic fibrosis (CF) is a complex genetic disease characterised by a progressive deterioration of lung function, which correlates with increased morbidity and mortality.^1 2^ The wide phenotypic variability observed in people with CF is only partly attributable to the diversity of variants identified in the Cystic Fibrosis Transmembrane Conductance Regulator (CFTR) gene. Although non-genetic factors, including environmental exposures, play a role in disease progression and severity, few studies have so far explored these associations, particularly in the UK.

Many systematic reviews of associations between air pollution and other respiratory diseases, such as asthma and chronic obstructive pulmonary disease, have confirmed the negative short-term and long-term impact of air pollution on the health of patients.^3 4^ In contrast, there have only been a handful of epidemiological studies in CF looking at associations between air pollutants and clinical outcomes, including pulmonary exacerbations, lung function and airway pathogens.^5–8^ A recent review highlighted important variability across studies due to differences in design and exposure assessment methods.^9^ Goss *et al* reported in a US study (n=11 484) that each 10 µg/m³ increase in particulate matter with a diameter of less than 2.5 μm (PM₂.₅) was associated with 21% higher odds of experiencing two or more pulmonary exacerbations, along with an average decline of approximately 24 mL in forced expiratory volume in 1 s (FEV_₁_).^7^ In a study conducted in São Paulo, Brazil (n=103), Farhat *et al* observed that a 45 µg/m³ increase in O₃ nearly doubled the risk of respiratory exacerbations among children and adolescents with CF (relative risk=1.86; 95% CI 1.14 to 3.02).^5^ In a multicentre retrospective case-crossover study conducted in Belgium (n=215), Goeminne *et al* found that short-term increases in PM₁₀, nitrogen dioxide (NO_₂_) and O_₃_ were associated with higher odds of antibiotic-treated pulmonary exacerbations (OR=1.034; 95% CI 1.003 to 1.067).^6^ Finally, in a 5-year retrospective study in Los Angeles (n=145), Jassal *et al* did not find significant links between ambient PM or O₃ concentrations and exacerbations. Instead, they reported substantially elevated exacerbation rates (OR=6.7; 95% CI 1.23 to 54.49) among patients living near major arterial roadways and concluded that traffic-related exposures, such as NO_2_, may be an important contributing factor.^8^

Despite the lack of specific scientific evidence on CF, air pollution and the quality of the local residential environment have a substantial impact on decisions made by people with CF in the UK. A recent survey of the financial burden of CF showed that 31% of adults with CF in the UK had made a decision to move house in search of somewhere with better air quality or a better environment.^10^

The UK Cystic Fibrosis Registry (CFR) is a high-quality national database of routine longitudinal health data of people with CF in England, Wales, Scotland and Northern Ireland.^11^ The UK CFR is predominantly based on annual reviews and represents one of the largest national CF datasets outside the USA.^11^ In parallel, the London Air Quality Network, which uses accurate automatic monitoring stations across the Greater London area, provides robust air pollution measurements, combined with high-resolution modelling.^12^ It provides a valuable data source that represents the estimate of ambient air pollution exposure of the local populations. Similar high-resolution models are being developed for the rest of the UK. The combination of the UK CFR and high-resolution modelled exposure data for London, therefore, offers a great opportunity to improve our understanding of the impact of outdoor air pollutants on people with CF.

This study used high-quality clinical data, alongside high-resolution exposures, to investigate the impact of two main air pollutants, PM_2.5_ and NO_2_, on lung function in people with CF residing in London over a period of 10 years (2008–2017). Understanding the relationship between air pollution and lung function decline in people with CF can provide valuable insights for clinical practice and public health interventions. Our hypothesis was that the detrimental effects of air pollution on lung function for people with CF living in London are more severe if they are exposed to higher levels of air pollutants (PM_2.5_ and NO_2_). The findings have the potential to inform disease management strategies, guide targeted treatments and patients’ and carers’ decisions, and shape public health initiatives aimed at improving respiratory health outcomes and overall well-being among people with CF in London and beyond.

## Methods

### Study design and participants

We conducted a retrospective longitudinal cohort study of data collected prospectively by the UK CFR on all registered people with CF in London with repeated measurements of the FEV_1_ between 2008 and 2017. These measurements were used to estimate the rate of decline in the percent predicted FEV_1_ (ppFEV_1_) in people with CF with a residential postcode in London in 2017. For this study, London is defined as the Greater London area, which is delimited by the boundaries of the 32 London boroughs and covers the largest, most populated city in the UK (8.9 million people; 13% of the national population). Among these people with CF, we excluded those under the age of 6 years in 2008 because of the lack of reliable standard pulmonary function tests in young children.^13^ Each individual matching our inclusion criteria (online supplemental figure S1) was geolocated based on their full residential postcode (postcode unit) recorded for each year of the study. In England and Wales, a postcode unit has an average of 43 (SD=38, median=33) residents and 18 (SD=15, median=14) occupied households. UK CFR data accessible to the authors for this study were fully anonymised, stored and processed on a secure server with restricted and controlled access in compliance with Registry requirements and UK data confidentiality and privacy laws. Coordinates of postcode units were obtained from the Ordnance Survey Code-Point Open dataset. Repeated annual records from the UK CFR allowed us to account for the residential history of people with CF. Although postcodes are regularly checked and updated in the UK CFR, some were missing for our study population. Missing postcodes were imputed based on a process detailed in the online supplemental methods S1 and summarised in online supplemental table S1.

### Health and air pollutant exposure data

FEV_1_ measurements in the UK CF Registry are performed at clinical sites using standard clinical devices during a stable period (ie, not an exacerbation), both throughout the year and at the annual review. Measurements are conducted with standardised spirometry techniques according to Registry procedures aligned with national and international guidelines. Further details on data source, coverage and quality measures of the Registry are fully described in Taylor-Robinson *et al*.^11^

FEV_1_ measurements recorded in the UK CFR were standardised to ppFEV_1_ based on age, sex and height using the GLI-2012 equations with the *rspiro* R package. Percent predicted FEV₁ was chosen as our primary outcome due to its long-established clinical relevance and widespread use in CF research, reflecting its robust association with disease progression, treatment response and survival. FEV₁ is the most consistently recommended and utilised measure for assessing lung function and response to interventions in CF.^14^ The ppFEV_1_ values were considered as a continuous variable in all our analyses.

To assign air pollutant exposure to people with CF within London, we used high-resolution modelled concentrations for PM_2.5_ and NO_2_ from the London Air Pollution Toolkit for each year of our study period. The Toolkit model, which is evaluated against data from the local reference monitoring network (www.londonair.org.uk), estimates air pollution concentrations across London with a resolution of 20 m by 20 m by incorporating detailed topography, meteorology and emissions data, and combining modelling-measurement and kernel-modelling approaches to predict the dispersion of air pollutants.^12^ The model is a bottom-up emissions inventory model, which incorporates full consideration of variations in meteorological conditions, including temperature and season. Detailed quality assurance and quality control procedures for this toolkit have been described previously.^15^ Mean exposure concentrations were extracted for the year (365 days) preceding the annual review date for all people with CF. Comparisons of the characteristics of people with CF with missing exposure for PM_2.5_ and NO_2_ are presented in online supplemental table S2 and S3, respectively.

### Covariates, confounders, effect modifiers and mediators

We considered age, sex and height as covariates; weight, CFTR genotype and *Pseudomonas aeruginosa* as potential mediators; socioeconomic status as a potential confounder; and CFTR modulator treatment and antibiotic use as potential effect modifiers (online supplemental figure S1).^13 16^ For the CFTR genotype, we devised a new eight-group classification based on the different combinations of the recognised terms developed in parallel with new CFTR modulating therapies: residual function (RF), minimal function (MF) and gating function.^17^ We developed this as a more precise classification system compared with other simpler classifications. A detailed description of this classification system was published in Khan *et al*.^17^ We classified the following as severe genotypes: homozygous *F508del*, *F508del*/MF, Gating/any (except RF mutation) or MF/MF. Infections with *P. aeruginosa* were defined by a positive culture in the year preceding the annual review. Positive cultures were defined as the sum of ‘chronic’ and ‘intermittent’. The UK CF Registry definition of ‘chronic’ is three or more isolates in the 12 months preceding the annual review. Infections are classified as ‘intermittent’ if there was at least one culture with positive growth over the same period (online supplemental table S4). We also accounted for the use of CFTR modulator treatments and inhaled antibiotics, which are two treatments that could substantially affect the lung function of people with CF. Although CFTR modulator therapies are commonly used now, their availability and use were much more limited during our study period. As a proxy of the socioeconomic status of people with CF, we used the national level 2011 UK Townsend deprivation score quintiles at the output area level (the highest resolution available), which are accessible in open access from the UK Data Service. The first quintile (Q1) refers to the least deprived areas of the UK, whereas the fifth quintile (Q5) corresponds to the most deprived. Links to the datasets and reference for the statistical software used are included in the online supplemental bibliography.

### Statistical analysis

A descriptive analysis was performed to explore the distribution of the demographic and clinical characteristics of people with CF. Correlation analyses (Pearson coefficient, r) were conducted to explore potential collinearity between our independent variables (online supplemental table S5). The mean, SD and range were reported for ppFEV_1_, PM_2.5_, NO_2_ and frequencies/proportions for age, sex, genotype, number of *P. aeruginosa* positive cultures and deprivation. To investigate the relationship between PM_2.5_ and NO_2_ exposures and the ppFEV_1_ decline in people with CF, we used a tertile-based approach that captured the most frequent exposures experienced by each person with CF throughout our 10-year study period (2008–2017). First, we divided the annual average PM_2.5_ or NO_2_ values into three equal groups (tertiles) based on their distribution: low, medium and high exposure. We then assigned each person with CF to a specific tertile (low, medium or high) based on their exposure profile (online supplemental methods S2).

Longitudinal analysis was performed using linear mixed models with random intercepts to estimate the decline in ppFEV_1_ over time associated with air pollutant exposure by including a dummy interaction term between air pollutant and time (follow-up year of the annual review).^18^ The model was adjusted for sex, CFTR genotype and deprivation, as well as for age, height, weight and *P. aeruginosa* positive culture (percentage of positive chronic, positive intermittent, negative), which were considered as time-varying variables. Different combinations of adjustments were tested using a forward approach, that is, adding one variable at a time. The Akaike information criterion (AIC) was used to compare model fit and guide variable selection, ensuring that the included variables best captured the association between air pollution exposure and lung function. The AIC values for the PM_2.5_ main model are summarised in online supplemental table S6. Additionally, variables with established biological and clinical relevance were retained, providing a robust analysis of the factors influencing ppFEV_1_. The coefficient estimates are reported as a percentage change in ppFEV_1_ with a 95% CI for PM_2.5_ and NO_2_ exposure levels. All comparisons used the 2008 low-exposure group as the reference category.

We conducted a series of sensitivity analyses to assess the consistency and robustness of our results (online supplemental table S7). First, we looked at a subset of people with CF whose residential postcode did not change during the study period (n=110). Second, we conducted stratified analyses by age group and CFTR genotype to explore potential modifying effects. Third, we used a simpler, more traditional classification of the CFTR genotype (homozygous *F508del*, heterozygous *F508del* and other) to test the influence of our new classification on our findings. Fourth, we excluded individuals who received CFTR modulator therapy during the study period. Although the availability and use of the CFTR modulators during our study period were limited, these treatments have such a substantial impact on people with CF that they needed to be considered. Finally, we compared individuals who used inhaled antibiotics *vs* those who did not.

All statistical analyses were performed using R V.4.4.1 on the UK Small Area Health Statistics Unit secure research systems at Imperial College London. Mixed-effect models were fitted using the ‘*lme4’* R package. All results were considered statistically significant for p<0.05.

## Results

Out of the 8965 people with CF who had an annual review in 2016 recorded in the UK CFR, a subset of 527 (5.9%) had a residential postcode in London. Of these, a total of 134 were excluded from this analysis for the following reasons: 114 were ≤14 years in 2017, that is, under 6 years old in 2008; 15 had a lung transplant and 5 had missing exposure data (incorrect postcode) (online supplemental figure S2). As a result, 393 people with CF (122 children and 271 adults) were included in our analysis. A further 95 and 39 people with CF could not be assigned to a specific exposure tertile for PM_2.5_ and NO_2_, resulting in 298 and 354 individuals being used, respectively, in the linear mixed models.

To assess the representativeness of this cohort of people with CF, a comparison of the demographic and clinical characteristics of people with CF with a residential postcode in London and of all people with CF in the UK CFR in 2016 is presented in table 1. The proportions of children (6–15 years old) and adults (≥16) were comparable (p=0.775). The proportion of females was significantly higher in London compared with the rest of the UK (p=0.048). The CFTR genotype composition among the two groups was also quite different (p<0.001). There was no statistically significant difference in the genotype classification using the simplified classification of genotype based purely on the *F508del* mutation (p=0.200). In the London people with CF study cohort in the UK, the proportion of patients with *P. aeruginosa* culture positivity rose from 41.7% in 2008 to 62.6% in 2011, remaining above 56% thereafter up to 2017. In contrast, among all people with CF in the UK, prevalence was stable at around 50%–53% until 2013, before declining to 43.9% in 2017. Significant differences between the two cohorts were observed in 2008, 2011–2012 and 2014–2017 (p<0.05). Although the distribution of people with CF by deprivation quintiles was fairly even across the UK, there was a clear overrepresentation of people with CF in the most deprived quintile in London (43.5%) and an underrepresentation in the two least deprived quintiles (5.9% in Q1 and 8.7% in Q2). Table 2 shows the summary statistics of preannual review estimated concentration of PM_2.5_ and NO_2_ by exposure tertile at the residential postcode of the study cohort of people with CF.

**Table 1:** Characteristics of people with CF in London and the UK

	People with CF study cohort in London n=393	People with CF in the UK n=8965	P value
Characteristics	n (%)	n (%)	
Age (years)			0.775
Children (6–15)	122 (31.0)	2268 (30.4)	
Adults (≥16)	271 (69.0)	5203 (69.6)	
Sex			**0.048**
Female	203 (51.7)	4175 (46.7)	
Male	190 (48.3)	4790 (53.3)	
Genotype			
New classification (8 categories)			**<0.001**
Homozygous F508del	189 (48.1)	4456 (49.7)	
F508del/MF	62 (15.8)	1757 (19.6)	
RF/other	32 (8.1)	980 (10.9)	
F508del/other	34 (8.7)	631 (7.0)	
Gating/any*	28 (7.1)	555 (6.2)	
MF/MF	23 (5.9)	291 (3.3)	
Other/other	13 (3.3)	157 (1.8)	
MF/other	12 (3.1)	138 (1.5)	
Traditional classification (3 categories)			**0.200**
Homozygous F508del	189 (48.1)	4456 (49.7)	
Heterozygous F508del	96 (24.4)	2388 (26.6)	
Other	108 (27.5)	2121 (23.7)	
*Pseudomonas aeruginosa* culture positive (chronic and intermittent)			
2008	164 (41.7)	3123 (51.3)	**0.014**
2009	190 (48.3)	3707 (50.3)	0.611
2010	225 (57.3)	4210 (53.0)	0.204
2011	246 (62.6)	4605 (53.1)	**0.004**
2012	238 (60.6)	4653 (52.9)	**0.022**
2013	223 (56.7)	4491 (49.6)	**0.045**
2014	234 (59.5)	4622 (49.0)	**0.002**
2015	223 (56.7)	4502 (47.0)	**0.006**
2016	241 (61.3)	4277 (44.1)	**<0.001**
2017	242 (61.6)	4337 (43.9)	**<0.000**
Census output area deprivation quintile (Townsend index)			**<0.001**
Q1 (least deprived)	23 (5.9)	1855 (20.7)	
Q2	34 (8.7)	1875 (20.9)	
Q3	85 (21.6)	1863 (20.8)	
Q4	80 (20.4)	1784 (19.0)	
Q5 (most deprived)	171 (43.5)	1588 (17.7)	

Comparison of characteristics of the study cohort of people with CF with a residential postcode in London and all people with CF in the UK CF Registry in 2016.

Other: Either the mutation was not identified, or it was identified but classification into one of the other categories was not possible.

* Except RF mutation.

CF, cystic fibrosis; MF, minimal function; RF, residual function.

**Table 2:** Air pollution exposure in people with CF in London

Year	n	Mean±SD	Median	Exposure tertile		
				Low	Medium	High
				Mean (Min, Max)	Mean (Min, Max)	Mean (Min, Max)
PM_2.5_ yearly average concentration before the annual ppFEV_1_ measurement						
2008	364	13.98±1.1	13.81	14.02 (11.68, 16.35)	13.94 (11.96, 16.27)	13.96 (11.68, 16.35)
2009	327	13.81±1.1	13.7	13.72 (11.64, 16.10)	13.87 (11.64, 16.69)	13.96 (11.92, 15.73)
2010	342	13.84±1.2	13.86	13.59 (11.64, 16.81)	13.88 (11.90, 16.02)	14.08 (12.11, 15.98)
2011	335	14.02±1.2	13.93	13.87 (11.96, 16.73)	14.16 (11.96, 16.43)	14.07 (12.07, 16.43)
2012	334	14.01±1.2	14.01	13.95 (11.51, 16.83)	13.97 (11.51, 16.34)	14.27 (11.89, 16.23)
2013	327	13.99±1.1	13.83	13.8 (12.05, 16.94)	14.11 (12.05, 16.79)	14.18 (12.04, 16.49)
2014	350	13.93±1.1	13.77	13.77 (12.04, 16.54)	13.99 (12.04, 17.33)	14.33 (12.40, 16.78)
2015	346	13.65±1.1	13.43	13.35 (11.73, 15.64)	13.72 (12.02, 16.59)	14.00 (12.10, 16.24)
2016	339	13.63±1.1	13.43	13.51 (11.44, 15.65)	13.62 (11.47, 15.90)	13.88 (11.47, 15.64)
2017	353	13.77±1.1	13.58	13.47 (11.92, 15.91)	13.81 (11.97, 16.13)	14.02 (12.10, 16.11)
NO_2_ yearly average concentration before the annual ppFEV_1_ measurement						
2008	309	36.16±5.4	35.5	31.48 (25.98, 44.61)	35.88 (28.65, 43.94)	42.49 (26.88, 53.16)
2009	315	38.29±5.7	37.71	33.05 (26.64, 39.22)	38.45 (32.49, 47.00)	45.31 (29.10, 56.71)
2010	323	36.60±5.4	35.84	31.58 (25.38, 38.17)	36.67 (30.54, 44.73)	43.07 (32.28, 54.20)
2011	334	33.23±5.0	32.71	28.45 (22.87, 35.46)	33.18 (28.36, 37.15)	39.17 (28.44, 49.18)
2012	337	36.18±5.1	35.73	31.16 (25.30, 39.30)	36.1 (31.99, 41.08)	42.09 (34.77, 52.24)
2013	345	34.54±5.3	33.84	29.31 (22.84, 38.91)	34.51 (30.70, 39.94)	40.47 (27.76, 50.60)
2014	350	34.33±5.2	33.68	29.26 (22.84, 38.22)	34.3 (28.88, 39.94)	40.36 (27.76, 55.47)
2015	355	34.58±5.1	34.11	29.83 (24.33, 51.63)	34.44 (26.78, 42.80)	40.07 (28.72, 51.25)
2016	370	34.50±4.9	34.1	29.53 (24.33, 36.31)	34.53 (30.03, 42.80)	39.66 (28.72, 48.42)
2017	379	34.64±4.9	34.12	29.62 (24.33, 39.90)	34.63 (30.03, 42.80)	39.68 (28.72, 48.42)

Summary of yearly preannual review estimated concentration of PM_2.5_ and NO_2_ at the residential postcode of the study cohort of people with CF in London between 2008 and 2017.

CF, cystic fibrosis; NO_2_, nitrogen dioxide; PM_2.5_, particulate matter with a diameter of less than 2.5 μm; ppFEV_1_, predicted forced expiratory volume in 1 s.

Correlation coefficients (r) between deprivation and PM_2.5_, NO_2_, weight and *P.aeruginosa* were all below 0.300 (online supplemental table S3); therefore, further exploration of cross-correlations was not explored.

Within our cohort of people with CF in London, the mean ppFEV_1_ decreased from 73.3% to 63.1% over the 10-year study period, corresponding to an average annual decrease of 1.1% (online supplemental figure S3). Within the adult subgroup, the mean ppFEV_1_ declined from 70.0% to 60.1% (ie, 0.99% per year), while in children, the mean ppFEV_1_ decreased from 81.5% to 69.6% (ie, 1.19% per year).

**Figure 1:**
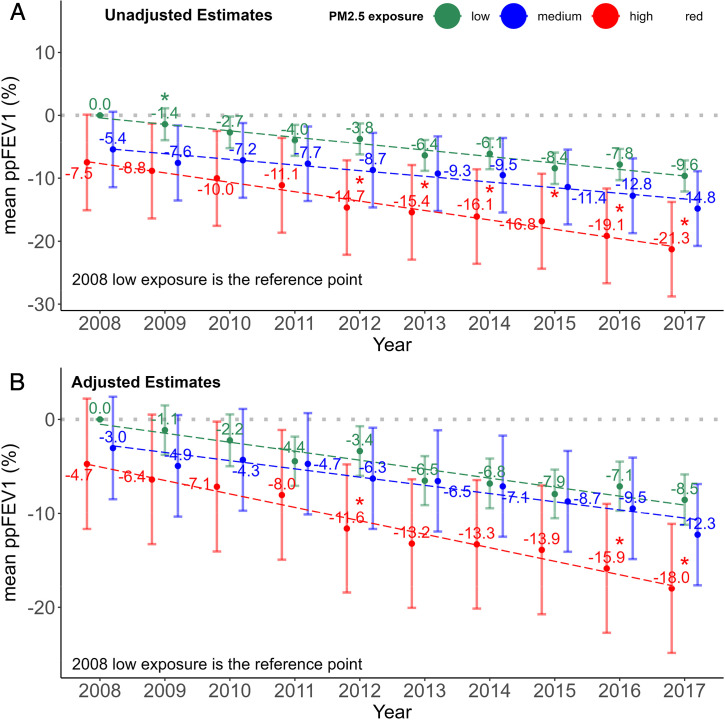
Lung function decline in people with CF based on PM_2.5_ exposure in London. Unadjusted (**A**) and adjusted models (**B**) of the estimated change in mean ppFEV_1_ percentage points by exposure tertile to PM_2.5_ among people with CF with a residential postcode in London between 2008 and 2017 (n=298). The model is adjusted for age at baseline, sex, genotype, height, weight, *Pseudomonas aeruginosa* and deprivation (Townsend). Number of people with CF: low=91, medium=126, high=81. CF, cystic fibrosis; PM_2.5_, particulate matter with a diameter of less than 2.5 μm; ppFEV_1_, predicted forced expiratory volume in 1 s.

In our adjusted linear mixed model for PM_2.5_ over the study period, the mean decline in absolute difference in ppFEV_1_ was −13.3% (95% CI –24.1% to –4.0%) in the high exposure group compared with those assigned to the low exposure group, which was −8.5% (95% CI −11.7% to −6.4%) by 2017 (figure 1).

A summary of our subgroup and sensitivity analyses is presented in online supplemental table S3. First, a subgroup analysis of people with CF (n=110, ppFEV_1_ measurements=1225) whose postcode did not change during the study period also showed a decline in the absolute difference in ppFEV_1_ between high and low PM_2.5_ exposure levels, consistent with the main findings over the study period. The magnitude of the decline of ppFEV_1_ was higher at 10.5% (95% CI −27.8% to −5.6%) in the high exposure group compared with 6.2% (95% CI −12.1% to −2.2%) in the low exposure group by 2017 (online supplemental figure S4).

Second, the overall decline in ppFEV_1_ was similar between CF children exposed to high and low levels of PM_2.5_. By 2017, the high-exposure group had experienced a decline in the absolute difference of 12.1% (95% CI −34.3% to −13.4%), while the low-exposure group had a similar decline of 13.2% (95% CI −20.5% to −8.6%) over the study period (online supplemental figure S5). The decline in adjusted ppFEV_1_ among CF adults was consistent with our primary findings. CF adults with high PM_2.5_ exposure experienced an average decline in the absolute difference of 13.1% (95% CI −21.3% to −3.81%) in adjusted ppFEV_1_, compared with 6.7% (95% CI −11.0% to −4.37%) in the low exposure group over the study period (online supplemental figure S6).

Finally, in our adjusted model, the analysis of people with CF with severe genotypes revealed a sharper decline in absolute difference mean ppFEV1 of 18.3% over our study period in the high exposure group (95% CI −24.3 to −2.7) compared with the 9.2%, low exposure group (95% CI −12.6 to −6.9) (online supplemental figure S7). The difference between these two groups was not statistically significant (p=0.105) (online supplemental figure S7).

**Figure 2:**
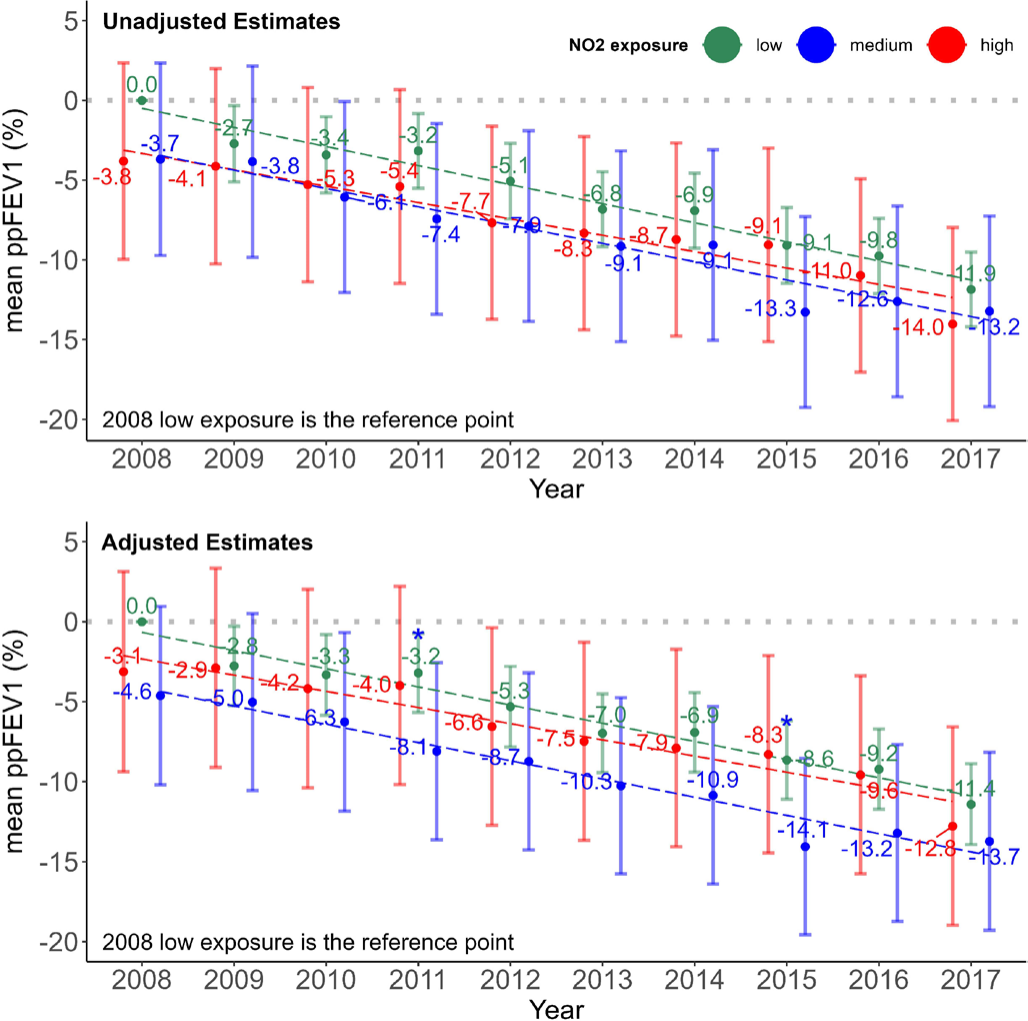
Lung function decline in people with CF based on NO_2_ exposure in London. Estimated change in mean ppFEV_1_ associated with annual exposure to NO_2_ with trend line among people with CF with a residential postcode in London between 2008 and 2017 (n=354). The model is adjusted for age at baseline, sex, genotype, height, weight, *Pseudomonas aeruginosa* and deprivation (Townsend). Number of people with CF: low=128, medium=116, high=110. CF, cystic fibrosis; NO_2_, nitrogen dioxide; ppFEV_1_, predicted forced expiratory volume in 1 s.

Using a similar approach, we did not find evidence of an association between ppFEV_1_ and exposure to NO_2_ concentration in people with CF in London (figure 2). In our adjusted linear mixed model for NO_2_, the mean decline in the absolute difference in ppFEV_1_ was −9.7% in the high exposure group compared with those assigned to the low exposure group, which was −11.4% over the study period. Subgroup and sensitivity analyses for NO_2_ are shown in online supplemental figures S8–S15.

## Discussion

This study investigated the effect of ambient PM_2.5_ and NO_2_ exposure on the decline of ppFEV_1_ among people with CF residing in London over a decade from 2008 to 2017. Using high-quality data from the UK CFR, the study included people with CF with a residential postcode in London and employed a high-resolution modelled exposure of PM_2.5_ and NO_2_. The findings suggest an association between high annual mean PM_2.5_ exposure and a higher rate of decline in ppFEV_1_ among people with CF, providing novel evidence about the impact of air pollutants on the health of people with CF in the UK.

We found that the distribution of people with CF by deprivation quintiles was fairly even across the UK. However, there were striking differences in the socioeconomic distribution of people with CF living in London. Specifically, 44% of people with CF were residents in the most deprived quintile, while only 6% and 9% lived in the least deprived quintiles (Q1 and Q2, respectively). This disparity could suggest that people with CF from less deprived backgrounds may have the means to move out of London to areas with better living conditions, while those in the most deprived quintile face barriers that prevent them from relocating. Many studies have demonstrated a strong association between low socioeconomic status and adverse health outcomes in people with CF.^19 20^ Taylor-Robinson *et al* reported that children with CF from socioeconomically deprived areas in the UK had significantly lower lung function compared with those from less deprived areas, even after adjusting for disease severity.^19^ Similarly, Stephenson *et al* found that people with CF from lower socioeconomic backgrounds in the USA experienced higher rates of pulmonary exacerbations, hospitalisations and missed treatments, contributing to poorer lung function and nutritional status.^20^ These findings highlight the persistent socioeconomic inequalities faced by people with CF from disadvantaged backgrounds, which may be attributed to factors such as delayed diagnosis, limited access to specialist care and barriers to adherence to treatments. Considering that the socioeconomic inequalities evidenced here were important within London but not nationally, further work is needed to confirm these findings. Specific interventions might be needed to support people with CF living in deprived urban areas. Given these marked socioeconomic disparities, findings from London may not fully generalise to CF populations in less deprived or more rural settings.

Lung function is a key prognostic marker in people with CF, and the decline in ppFEV_1_ over time plays an important role in monitoring CF disease progression, guiding treatment based on responses to therapy and improving overall clinical outcomes in people with CF.^21^ In our analysis, we found a 1.08% (95% CI −1.21% to −0.95%) annual average decline in ppFEV_1_ among people with CF residing in London. This decline is lower than the range reported in previous studies on adult CF populations, where lung function annual decline rates varied between 1.5% and 2.0%.^22 23^ This difference may be due to the duration of our study, which was significantly longer than the follow-up periods in the previous studies. In the context of general pollution in the UK, the annual decline in FEV₁ varies by age and sex, but is generally estimated at approximately 18–46 mL/year, with long-term cohort studies reporting a median decline of around 22 mL/year.

Understanding the effects of air pollutants on lung function decline is essential for implementing effective strategies to improve the overall health of people with CF. In line with our hypothesis, this longitudinal analysis suggested a faster decline in ppFEV_1_ among high PM_2.5_ levels compared with low levels of PM_2.5_ exposure in people with CF. Mirroring the main analysis, a consistent trend was seen in the geographically stable CF population (ie, the subgroup of people with CF who did not change their postcode). This reinforces the hypothesis that reduced air pollution exposure benefits lung function in CF. A previous study, conducted in the USA, which looked at the relationship between PM_2.5_ and lung function in people with CF in a large cohort (n=11 484), identified a 0.5% (95% CI 0.3% to 0.9%) reduction in ppFEV_1_ for every 10 µg/m^3^ increase in PM_2.5_.^7^ Despite methodological differences in the precision and classification of PM_2.5_ exposure, both investigations therefore suggest that PM_2.5_ has a negative effect on lung function. The implications of the results are important from both clinical and public health perspectives. The findings of this study stress the importance of lowering exposure to high levels of PM_2.5_ concentration to prevent a quicker decline in lung function in people with CF. This may be accomplished by raising knowledge of different guidelines, such as the air quality index in London and the WHO legal limit of PM_2.5_, which was recently revised to 5 µg/m^3^, and avoiding areas of high pollution by following guidance associated with these tools.

Our analysis did not identify a distinct association between ppFEV_1_ and NO_2_ exposure among people with CF in London. Epidemiological evidence on the effects of NO_2_ on lung function in people with CF is limited. To our knowledge, the study by Goss *et al* is the only other investigation on this topic, but it is a cross-sectional study.^7^ Consistent with our findings, their study did not identify a significant association between NO_2_ exposure and lung function decline in people with CF.^7^ Evidence of an association between NO_2_ and ppFEV_1_ has been inconsistent in non-CF populations.^24 25^ In our cohort, the lack of association with NO₂ may reflect limited spatial contrasts in London,^26^ strong correlations with other traffic pollutants^27^ and greater potential for exposure misclassification due to steep roadside gradients^28^ and short-term variability.^29^ These factors can bias associations toward the null.^30^ By contrast, PM₂.₅ captures a broader mix of sources, which may explain its clearer signal. The absence of an association is somewhat surprising, given the relevance of NO_2_ exposure to respiratory health and the known susceptibility of people with CF to airway complications.^7 31 32^ This highlights the need for further research to comprehensively investigate the interplay between respiratory function, environmental factors and geographic location in people with CF residing in urban environments.

We found limited evidence of a differential effect of PM₂.₅ on ppFEV_1_ in children, as declines were comparable between high-exposure and low-exposure groups. The differences between children (6–15 years) and adults (≥16) may reflect age-related variations in disease progression, lung growth and cumulative environmental exposures.^33^ Children with CF generally have higher baseline lung function, but their developing lungs may be more susceptible to the adverse effects of air pollution, leading to greater proportional declines.^34^ Adults, in contrast, may show smaller relative changes due to already reduced baseline lung function,^35^ survivor effects^36^ and the possibility of better access to targeted treatments over the study period.^37^ Further research with a larger sample size is warranted to investigate potential effects that may not have been detected in this study.

There was a significant association between PM_2.5_ exposure level and ppFEV_1_ among the subset of CF with a severe genotype. People with CF with a severe genotype tend to be sicker and therefore are more likely to have a worse decline in ppFEV_1_.^38^ The implications of these results highlight the importance of reducing PM_2.5_ exposure levels, particularly in people with CF with a severe genotype. Additionally, these results highlight the potential need for better information about residential PM_2.5_ levels for people with CF, as well as for their carers and clinicians. In addition, targeted and more intensive interventions for people with CF who have a severe genotype and live in areas with high and medium exposure to PM_2.5_ might need to be considered.

There is growing evidence that the association between air pollution and respiratory disease is not linear.^39^ This is reflected in our findings as the decline in ppFEV_1_ over time in the medium-exposure group did not fall between the values found in the high-exposure and low-exposure groups. Some of these differences might be explained by the relatively small sample size considered, particularly in some subgroup analyses. High-resolution exposure modelled surfaces for the whole of the UK would be required to enable a national-level study, making full use of the UK CFR data.

Apart from in 2008 and 2009, the proportion of people with CF with *P. aeruginosa* positive culture (chronic or intermittent) was higher in London than in the rest of the UK. On average, between 2010 and 2017, 59.5% of people with CF had a positive culture compared with 49.1% elsewhere. Although the proportion of positive cultures seemed to decline in the UK during that period, which is in line with previous observational studies,^40^ it remained fairly stable in London. This higher and more persistent prevalence in London may limit the generalisability of our results to regions with a lower *P. aeruginosa* burden.

A key strength of this study was the availability of repeated high-quality measurements of ppFEV_1_ and comprehensive individual postcode-level PM_2.5_ exposure data from a high-resolution model over the study period. Additionally, using a longitudinal research design improved statistical strength by reducing the likelihood of within-person variation over time and improving the statistical power to detect associations. The study design also helped to control time-variant confounding variables like age, height, weight and number of *P. aeruginosa* cultures, as well as reducing confounding bias, strengthening the study’s internal validity and increasing the ability to attribute observed effects to PM_2.5_ and NO_2_ exposure.

Outdoor PM_2.5_ and NO_2_ concentrations may not fully reflect individual exposures, as people with CF may spend significant periods indoors or away from home. There is a growing focus on indoor air quality and exposure in different places. More advanced technologies based on personal monitors are needed to assess individual exposure more accurately in future studies. A national-level study of the impact of air pollutants on people with CF would maximise the use of the large data available in the UK CFR Registry. Imputation methods may be used to reduce the number of people with CF excluded from such a study. Future work might favour the use of z-scores, rather than ppFEV_1_. Although ppFEV_1_ accounts for age, sex and height, the % predicted measure has been shown to be affected by some age-related and height-related bias and assumes variability proportional to the predicted value, particularly in cohorts spanning a wide age range. While ppFEV_1_ was chosen for consistency with clinical reporting standards in CF care and prior UK CF Registry studies, the potential bias of this measurement should be considered when interpreting our findings. Other spirometry indices (eg, FVC and FEF25-75), the rate of pulmonary exacerbations and mortality could be considered, alongside the impact of other pollutants, in particular ozone (O_3_), and lifestyle and occupational factors not available in the UK CF Registry data. A more in-depth study of the exposome in people with CF could also include various ‘omics’ datasets.

The levels of PM_2.5_ and NO_2_ used to be higher in London than in other cities and rural areas, but the implementation of strict interventions in large cities (eg, Ultra-Low Emission Zone since 2019 in London) has substantially reduced these gaps. Importantly, indoor exposure to PM_2.5_ and NO_2_ could potentially have a higher impact on health than ambient exposure. In this respect, our findings suggest that interventions to reduce exposure might be beneficial for people with CF anywhere across the UK, even if they receive a CFTR modulator treatment.

Overall, our results underline the need for patient-specific care plans accounting for environmental factors and emphasise the importance of continued research to better understand the factors that are significantly associated with a faster decline of FEV_1_ in people with CF. Understanding changes in ppFEV_1_ in people with CF is important, as early identification of a faster decline in ppFEV_1_ can inform treatment decisions and potentially help better manage the progression of the disease.

## Conclusions

This study found that higher annual mean PM_2.5_ exposure levels were associated with a greater decline in ppFEV_1_ in people with CF in London between 2008 and 2017. These findings provide valuable new evidence of the negative impact of PM_2.5_ exposure on people with CF. To fully understand the impact of PM_2.5_ exposure on lung function in people with CF, particularly regarding age-specific susceptibility and potential interactions with other factors, further research is needed. Studies with larger sample sizes, longitudinal designs, a focus on indoor PM_2.5_ exposure and consideration of daily activity patterns are particularly needed. Finally, although the introduction of CFTR modulator therapies has significantly improved outcomes for many people with CF, our study confirms that there is still a need for non-pharmacological interventions to ensure that health improvements are maximised regardless of drug treatment options available.

## Supplementary material

10.1136/thorax-2024-222710 online supplemental file 1

10.1136/thorax-2024-222710 online supplemental file 2

## Data Availability

Data are available on reasonable request.
