# Supplementary material for: Impact of air pollution on lung function in cystic fibrosis over a decade in London: a UK CF Registry study
Source: Thorax. 2026 Feb 5;81(9):e222710. doi: 10.1136/thorax-2024-222710 (PMC13479626; doi:10.1136/thorax-2024-222710)
Supplement: Supplementary file 1 [file thorax-81-9-s002.pdf]

Impact of air pollution on lung function in cystic fibrosis over a decade in London: a UK CF Registry study

Dr Muhammad Saleem Khan MSK *et al*. Thorax 2024. DOI: 10.1136/thorax-2024-222710

Directed acyclic graph (DAG). Associations between exposure, potential mediator and outcome in people with CF in the London, UK CF Registry. Exposures are shown in blue; outcomes in orange; mediators in green; effect modifiers in purple; covariates in white; and confounders in yellow. Grey boxes indicate variables not taken into account in our analysis.

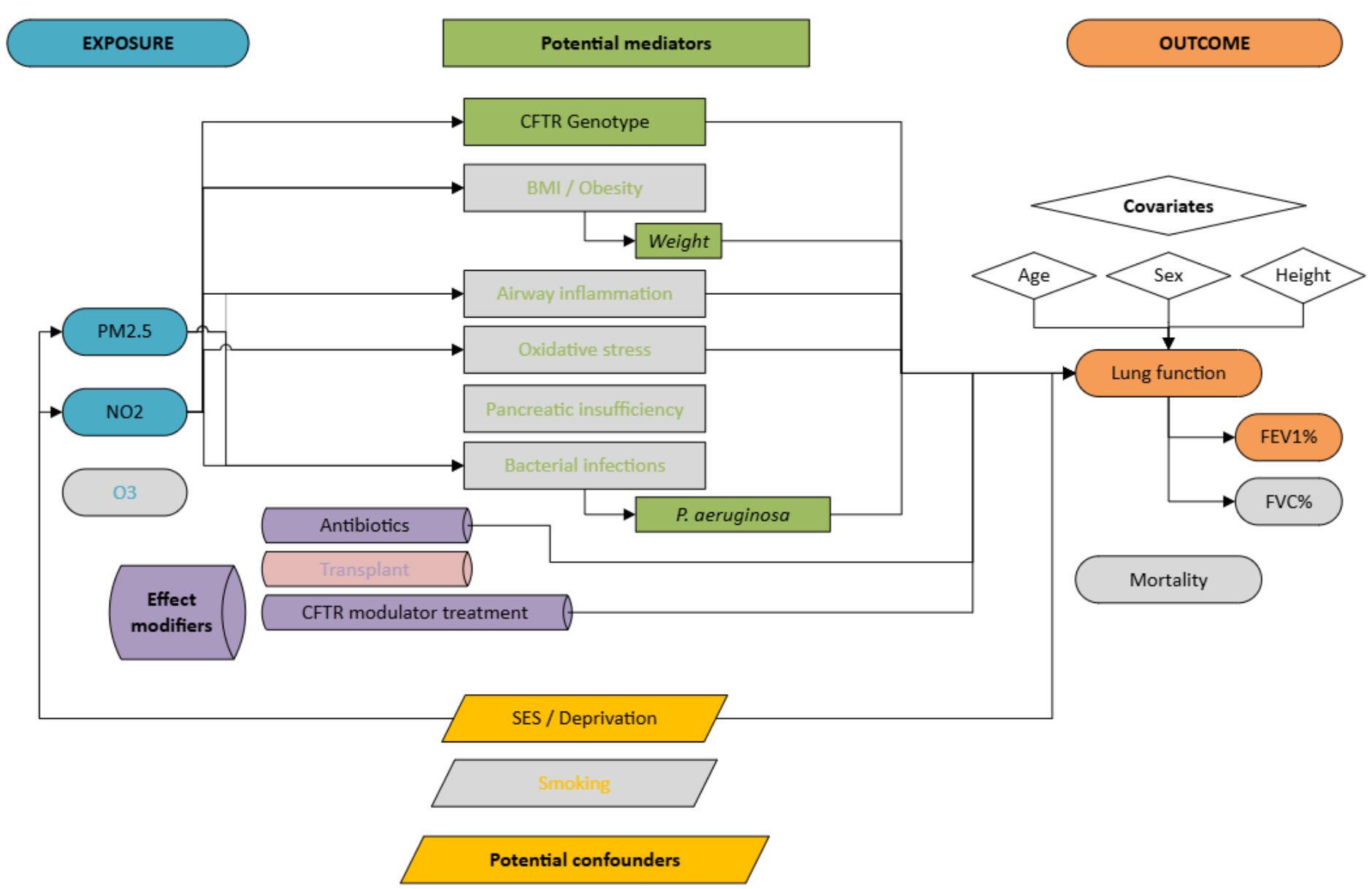

Figure **: Lung function decline in people with cystic fibrosis based on $PM_{2.5}$ exposure in London**

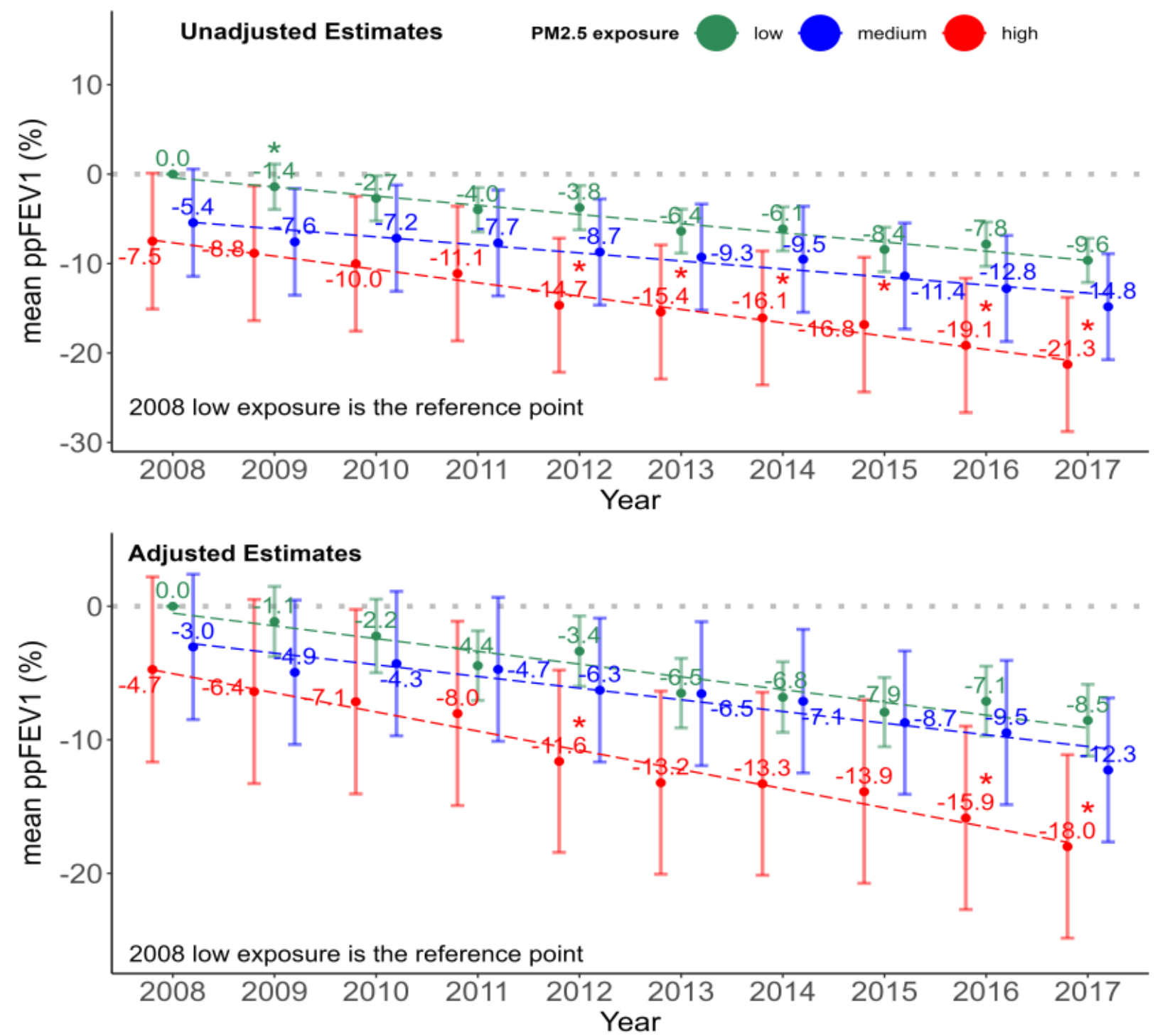

The model is adjusted for age at baseline, sex, genotype, height, weight, *Pseudomonas aeruginosa* and deprivation (Townsend).
Number of people with CF: low = 91, medium = 126 , high = 81.

Thorax thorax.bmj.com @ThoraxBMJ British Thoracic Society BMJ Group
