# Supplementary material for: Impact of air pollution on lung function in cystic fibrosis over a decade in London: a UK CF Registry study
Source: Thorax. 2026 Feb 5;81(9):e222710. doi: 10.1136/thorax-2024-222710 (PMC13479626; doi:10.1136/thorax-2024-222710)
Supplement: Supplementary file 2 [file thorax-81-9-s001.docx]

# Supplemental material

**Impact of air pollution on lung function in cystic fibrosis over a decade in London: a UK CF Registry study**

Muhammad Saleem Khan, Benjamin Barratt, Bethan Davies, Nicholas J. Simmonds and Frédéric B. Piel

Contents

[Supplemental material	1](#_Toc214628716)

[Supplementary Methods	4](#_Toc214628717)

[**Supplementary Methods S1**: Imputation criteria for replacing missing postcode	4](#_Toc214628718)

[**Supplementary Methods S2**: Exposure classification	4](#_Toc214628719)

[Supplementary Tables	5](#_Toc214628720)

[**Supplementary Table S1**: **Missing postcodes**. Explanation of the strategy for replacing a missing postcode	5](#_Toc214628721)

[**Supplementary Table S2**: Comparison of demographic and clinical characteristics of people with CF with and without PM_2.5_ exposure data	6](#_Toc214628722)

[**Supplementary Table S3**: Comparison of demographic and clinical characteristics of people with CF with and without NO_2_ exposure data	7](#_Toc214628723)

[**Supplementary Table S4: Pseudomonas aeruginosa culture status**. Culture status among people with cystic fibrosis in London between 2008 and 2017, based on data from the UK Cystic Fibrosis Registry	8](#_Toc214628724)

[**Supplementary Table S5**: **Correlation analysis**. Correlation between the exposure (air pollution) and socioeconomic status, and between socioeconomic status and weight and Pseudomonas aeruginosa	8](#_Toc214628725)

[**Supplementary Table S6**: **Model comparisons**. Value of the Akaike Information Criterion (AIC) for different regression model for PM_2.5_ with the addition of covariates using a forward approach	8](#_Toc214628726)

[**Supplementary Table S7**. **Summary of sensitivity analyses.** Differential change in ppFEV1 within people with CF in London in the high-, medium- and low-exposure tertile sub-groups for PM2.5 and NO2 in London, over our study period (2008-2017). Percentage change and sample sizes are for adjusted analyses accounting for age, sex, weight, CFTR genotype, P. aeruginosa and deprivation. Severe genotypes include homozygous F508del, F508del/minimal function, Gating/any (except residual function mutation) or minimal function/minimal function as described in Khan et al.21	9](#_Toc214628727)

[Supplementary Figures	10](#_Toc214628728)

[**Supplementary Figure S1**: **Directed acyclic graph (DAG).** Associations between exposure, potential mediator and outcome in people with CF in the London, UK CF Registry. Exposures are shown in blue; outcomes in orange; mediators in green; effect modifiers in purple; covariates in white; and confounders in yellow. Grey boxes indicate variables not taken into account in our analysis.	10](#_Toc214628729)

[**Supplementary Figure S2**: **Flow diagram.** Inclusion and exclusion criteria used for people with CF in the London, UK CF Registry	11](#_Toc214628730)

[**Supplementary Figure S3**: Overall mean ppFEV_1_ trend among people with CF with a residential postcode in London between 2008 and 2017 (n=393).	12](#_Toc214628731)

[**Supplementary Figure S4**: **Lung function and PM_2.5_ in CF “non-movers”**. Unadjusted (A) and adjusted (B) estimated change in mean ppFEV_1_ associated with annual exposure of PM_2.5_ among people with CF who did not change their residential postcode in London between 2008 and 2017 (n=110).	13](#_Toc214628732)

[**Supplementary Figure S5**: **Lung function and PM_2.5_ in CF children.** Unadjusted (A) and adjusted (B) estimated change in mean ppFEV_1_ associated with annual exposure of PM_2.5_ among children with CF (6 – 15 years) with a residential postcode in London between 2008 and 2017 (n=96).	14](#_Toc214628733)

[**Supplementary Figure S6**: **Lung function and PM_2.5_ in CF adults.** Unadjusted (A) and adjusted (B)estimated change in mean ppFEV_1_ associated with annual exposure of PM_2.5_ among adults with CF (≥16 years) with a residential postcode in London between 2008 and 2017 (n=202).	15](#_Toc214628734)

[**Supplementary Figure S7 ‎**: **Lung function and PM_2.5_ in severe CF.** Unadjusted (A) and adjusted (B) estimated change in mean ppFEV_1_ associated with annual exposure of PM_2.5_ among people with CF with a severe genotype in London between 2008 and 2017 (n=234).	16](#_Toc214628735)

[**Supplementary Figure S8‎**: **Lung function and PM_2.5_ in CF not on modulator.** Unadjusted (A) and adjusted (B) estimated change in mean ppFEV_1_ associated with annual exposure of PM_2.5_ among people with CF not on CFTR modulator treatment at any point between 2008 and 2017, in London	17](#_Toc214628736)

[**Supplementary Figure S9‎**: **Lung function and PM_2.5_ in CF on inhaled antibiotics.** Unadjusted (A) and adjusted (B) estimated change in mean ppFEV_1_ associated with annual exposure of PM_2.5_ among people with CF who received inhaled antibiotic treatment between 2008 and 2017, in London	18](#_Toc214628737)

[**Supplementary Figure S10:** **Lung function and NO_2_ in “CF non-movers”.** Unadjusted (A) and adjusted (B) estimated change in mean ppFEV_1_ associated with annual exposure of NO_2_ among people with CF who did not change their residential postcode between 2008 and 2017 (n=144).	19](#_Toc214628738)

[**Supplementary Figure S11**: **Lung function and NO_2_ in CF children”.** Unadjusted (A) and adjusted (B)estimated change in mean ppFEV_1_ associated with annual exposure of NO_2_ among children with CF (6 – 15 years) with a residential postcode in London between 2008 and 2017	20](#_Toc214628739)

[**Supplementary Figure S12**: **Lung function and NO_2_ in CF adults.** Unadjusted (A) and adjusted (B) estimated change in mean ppFEV_1_ associated with annual exposure of NO_2_ among adults with CF (≥16 years) with a residential postcode in London between 2008 and 2017 (n=240).	21](#_Toc214628740)

[**Supplementary Figure S13‎**: **Lung function and NO_2_ in severe CF.** Unadjusted (A) and adjusted (B) estimated change in mean ppFEV_1_ associated with annual exposure of NO_2_ among people with CF with a severe genotype in London between 2008 and 2017	22](#_Toc214628741)

[**Supplementary Figure S14‎**: **Lung function and NO_2_ in CF not on modulator.** Unadjusted (A) and adjusted (B) estimated change in mean ppFEV_1_ associated with annual exposure of NO_2_ among people with CF not on CFTR modulator treatment at any point between 2008 and 2017, in London	23](#_Toc214628742)

[**Supplementary Figure S15‎**: **Lung function and NO_2_ in CF on inhaled antibiotics.** Unadjusted (A) and adjusted (B) estimated change in mean ppFEV_1_ associated with annual exposure of NO_2_ among people with CF who received antibiotic treatment between 2008 and 2017, in London	24](#_Toc214628743)

[Supplementary References	25](#_Toc214628744)

### Supplementary Methods

#### **Supplementary Methods S1**: Imputation criteria for replacing missing postcode

Residential postcodes for all people with CF for each year between 2008 and 2017 were taken from the UK CF Registry's postcode dataset. Although postcodes are meant to be recorded and updated at annual reviews, we identified some gaps and inconsistencies. We needed to have all postcodes between 2008 and 2017 for all people with CF in this study to determine the PM_2.5_ exposure. To do this, we developed the following rules to impute the missing postcodes between 2008 and 2017 for all the people with CF.

i) A missing postcode was replaced with the following year's postcode if neighbouring postcodes were the same on both sides, for example, a postcode is missing for 2015, but the 2014 and 2016 postcodes were the same, so the missing postcode was replaced with the 2016 postcode.
ii) A missing postcode was replaced with the following year's postcode if neighbouring postcodes were different on both sides. See the below table for more details.
iii) Where two successive postcodes were missing, the first missing postcode was replaced with the previous year's postcode, and the second missing postcode was replaced with the following year's postcode.
iv) Where three postcodes in a row were missing, the first missing postcode was replaced with the previous year's postcode, the second missing postcode was replaced with the most common postcode during the study period, and the third missing postcode was replaced with the following year's postcode.
v) Where four postcodes in a row were missing, the first two missing postcodes were replaced with the previous year's postcode, 3^rd^ and 4^th^ missing postcodes were replaced with the following year's postcode.
vi) Where more than four postcodes were missing but not in a row, these were replaced using the above rule where appropriate.

#### **Supplementary Methods S2**: Exposure classification

To be classified as "low" exposure, a CF individual needed at least four annual average PM_2.5_ or NO_2_ values within the low tertile range for the pollutant considered. Similar conditions were applied for medium and high exposure tertiles: at least four annual average values within the respective tertile range for classification. The requirement of at least four measurements within each tertile range minimised the risk of misclassification of participants who may have experienced occasional fluctuations in exposure but primarily resided within a specific tertile group and provided a more accurate representation of the most frequent exposures experienced by each group.

### Supplementary Tables

#### **Supplementary Table S1**: **Missing postcodes**. Explanation of the strategy for replacing a missing postcode

| ID | 2008 | 2009 | 2010 | 2011 | 2012 | 2013 | 2014 |
| --- | --- | --- | --- | --- | --- | --- | --- |
| X | a | a | Missing ->a | Missing -> b | b | b | b |
| Y | e | Missing -> e | Missing -> e | Missing -> b | Missing -> b | b | b |
| Z | h | h | Missing -> h | g | g | Missing -> c | c |

The ID column represents a patient. Capital letters in columns 2008 to 2014 represent a postcode.

#### **Supplementary Table S2**: Comparison of demographic and clinical characteristics of people with CF with and without PM_2.5_ exposure data

| **Characteristic** | **Missing**  n = 95 | **Not missing**  n = 298 | **p-value^1^** |
| --- | --- | --- | --- |
| **Age (years)** |  |  | 0.374 |
| Children (6 – 15) | 69 (73%) | 202 (68%) |  |
| Adults (≥ 16) | 26 (27%) | 96 (32%) |  |
| **Sex** |  |  | 0.801 |
| Female | 48 (51%) | 155 (52%) |  |
| Male | 47 (49%) | 143 (48%) |  |
| **Genotype** |  |  |  |
| ***New classification (8 categories)*** |  |  | 0.360 |
| Homozygous F508del | 16 (17%) | 46 (15%) |  |
| F508del/MF | 8 (8.4%) | 26 (8.7%) |  |
| RF/other | 4 (4.2%) | 24 (8.1%) |  |
| F508del/other | 43 (45%) | 146 (49%) |  |
| Gating/any** | 5 (5.3%) | 18 (6.0%) |  |
| MF/MF | 4 (4.2%) | 8 (2.7%) |  |
| Other/other | 2 (2.1%) | 11 (3.7%) |  |
| MF/other | 13 (14%) | 19 (6.4%) |  |
| ***Traditional classification (3 categories)*** | | | 0.808 |
| Homozygous F508del | 24 (25%) | 72 (24%) |  |
| Heterozygous F508del | 43 (45%) | 146 (49%) |  |
| Other | 28 (29%) | 80 (27%) |  |
| **Census Output Area (COA) deprivation quintile (Townsend index)** | | | 0.196 |
| Q1 (least deprived) | 4 (4.2%) | 19 (6.4%) |  |
| Q2 | 13 (14%) | 21 (7.0%) |  |
| Q3 | 24 (25%) | 61 (20%) |  |
| Q4 | 18 (19%) | 62 (21%) |  |
| Q5 (most deprived) | 36 (38%) | 135 (45%) |  |
| ***P. aeruginosa* culture *positive*** |  |  | 0.353 |
| Negative | 57 (60%) | 172 (58%) |  |
| Positive - Chronic | 32 (34%) | 92 (31%) |  |
| Positive - Intermittent | 6 (6.3%) | 34 (11%) |  |
| ^1^Pearson's Chi-squared test | | | |

#### **Supplementary Table S3**: Comparison of demographic and clinical characteristics of people with CF with and without NO_2_ exposure data

| **Characteristic** | **Missing**  n = 39 | **Not missing**  n = 354 | **p-value^1^** |
| --- | --- | --- | --- |
| **Age (years)** |  |  | 0.134 |
| Children (6 – 15) | 31 (79%) | 240 (68%) |  |
| Adults (≥ 16) | 8 (21%) | 114 (32%) |  |
| **Sex** |  |  | 0.699 |
| Female | 19 (49%) | 184 (52%) |  |
| Male | 20 (51%) | 170 (48%) |  |
| **Genotype** |  |  |  |
| ***New classification (8 categories)*** |  |  | 0.733 |
| Homozygous F508del | 8 (21%) | 54 (15%) |  |
| F508del/MF | 3 (7.7%) | 31 (8.8%) |  |
| RF/other | 4 (10%) | 24 (6.8%) |  |
| F508del/other | 17 (44%) | 172 (49%) |  |
| Gating/any** | 1 (2.6%) | 22 (6.2%) |  |
| MF/MF | 0 (0%) | 12 (3.4%) |  |
| Other/other | 1 (2.6%) | 12 (3.4%) |  |
| MF/other | 5 (13%) | 27 (7.6%) |  |
| ***Traditional classification~~)~~ (3 categories)*** | | | 0.801 |
| Homozygous F508del | 11 (28%) | 85 (24%) |  |
| Heterozygous F508del | 17 (44%) | 172 (49%) |  |
| Other | 11 (28%) | 97 (27%) |  |
| ***P. aeruginosa* culture *positive*** |  |  | 0.502 |
| Negative | 22 (56%) | 207 (58%) |  |
| Positive - Chronic | 11 (28%) | 113 (32%) |  |
| Positive - Intermittent | 6 (15%) | 34 (9.6%) |  |
| **Census Output Area (COA) deprivation quintile (Townsend index)** | | | 0.967 |
| Q1 (least deprived) | 3 (7.7%) | 20 (5.6%) |  |
| Q2 | 3 (7.7%) | 31 (8.8%) |  |
| Q3 | 8 (21%) | 77 (22%) |  |
| Q4 | 7 (18%) | 73 (21%) |  |
| Q5 (most deprived) | 18 (46%) | 153 (43%) |  |
| ^1^Pearson's Chi-squared test | | | |

**Supplementary Table S4: Pseudomonas aeruginosa culture status**. Culture status among people with cystic fibrosis in London between 2008 and 2017, based on data from the UK Cystic Fibrosis Registry.

|  |  | **Year** | | | | | | | | | |
| --- | --- | --- | --- | --- | --- | --- | --- | --- | --- | --- | --- |
| ***P. aeruginosa*** |  | **2008** | **2009** | **2010** | **2011** | **2012** | **2013** | **2014** | **2015** | **2016** | **2017** |
| Positive culture - Chronic | **n** | 124 | 133 | 161 | 179 | 179 | 177 | 181 | 183 | 177 | 180 |
|  | ***%*** | *32%* | *34%* | *41%* | *46%* | *46%* | *45%* | *46%* | *47%* | *45%* | *46%* |
| Positive culture - Intermittent | **n** | 40 | 57 | 64 | 67 | 59 | 46 | 53 | 40 | 64 | 62 |
|  | ***%*** | *10%* | *15%* | *16%* | *17%* | *15%* | *12%* | *13%* | *10%* | *16%* | *16%* |
| Total positive | **n** | 164 | 190 | 225 | 246 | 238 | 223 | 234 | 223 | 241 | 242 |
|  | ***%*** | 42% | 49% | 57% | 63% | 61% | 57% | 59% | 57% | 61% | 62% |
| No positive culture | **n** | 229 | 203 | 168 | 147 | 155 | 170 | 159 | 170 | 152 | 151 |
|  | ***%*** | *58%* | *52%* | *43%* | *37%* | *39%* | *43%* | *40%* | *43%* | *39%* | *38%* |
| Total | N | 393 | 393 | 393 | 393 | 393 | 393 | 393 | 393 | 293 | 393 |

#### **Supplementary Table S5**: **Correlation analysis**. Correlation between the exposure (air pollution) and socioeconomic status, and between socioeconomic status and weight and Pseudomonas aeruginosa

| *Cross-correlation variable* | Pearson’s correlation coefficient |
| --- | --- |
| PM₂.₅ and deprivation (Townsend) quintiles | 0.068 |
| NO₂ and deprivation (Townsend) quintiles | 0.260 |
| Weight and deprivation (Townsend) quintiles | 0.015 |
| *P. aeruginosa* infection and deprivation (Townsend) quintiles | 0.237 |

#### **Supplementary Table S6**: **Model comparisons**. Value of the Akaike Information Criterion (AIC) for different regression model for PM_2.5_ with the addition of covariates using a forward approach

| Model | AIC |
| --- | --- |
| Interaction Year * Exposure | 27,868.6 |
| Interaction Year * Exposure + Age | 27,841.5 |
| Interaction Year * Exposure + Age + Sex | 27,788.5 |
| Interaction Year * Exposure + Age + Sex + CFTR Genotype | 27,775.5 |
| Interaction Year * Exposure + Age + Sex + CFTR Genotype + Height | 25,600.8 |
| Interaction Year * Exposure + Age + Sex + CFTR Genotype +Height + Weight | 25,561.0 |
| Interaction Year * Exposure + Age + Sex + CFTR Genotype +Height + Weight + *P. aeruginosa positive culture* | 25,561.5 |
| Interaction Year * Exposure + Age + Sex + CFTR Genotype+ Height + Weight + *P. aeruginosa* positive culture and deprivation *(Townsend index)* | 25,566.1 |

#### **Supplementary Table S7**. **Summary of sensitivity analyses.** Differential change in ppFEV1 within people with CF in London in the high-, medium- and low-exposure tertile sub-groups for PM2.5 and NO2 in London, over our study period (2008-2017). Percentage change and sample sizes are for adjusted analyses accounting for age, sex, weight, CFTR genotype, P. aeruginosa and deprivation. Severe genotypes include homozygous F508del, F508del/minimal function, Gating/any (except residual function mutation) or minimal function/minimal function as described in Khan et al.21

|  | *PM_2._*_5_ | | | | *NO_2_* | | | |
| --- | --- | --- | --- | --- | --- | --- | --- | --- |
|  | *All* | *Low* | *Medium* | *High* | *All* | *Low* | *Medium* | *High* |
| All people with CF |  | **-8.5%** | **-9.3%** | **-13.3%** |  | **-11.4%** | **-9.1%** | **-9.7%** |
| *n* | *298* | *91* | *126* | *81* | *354* | *128* | *116* | *110* |
| *p-value* |  | *0.181* | *0.096* | ***0.008*** |  | *0.556* | *0.354* | *0.556* |
| People with CF with the same postcode |  | **-6.2%** | **-8.1%** | **-10.5%** |  | **-10.4%** | **-10.4%** | **-5.8%** |
| *n* | *110* | *35* | *43* | *32* | *144* | *64* | *49* | *31* |
| *p-value* |  | *0.272* | *0.234* | ***0.034*** |  | *0.174* | *0.062* | *0.585* |
| Children (6 – 15 yrs) |  | **-13.2%** | **-6.1%** | **-12.3%** |  | **-11.0%** | **-10.9%** | **-12.7%** |
| *n* | *96* | *27* | *40* | *29* | *114* | *50* | *41* | *23* |
| *p-value* |  | *0.968* | ***0.011*** | ***0.014*** |  | *0.869* | *0.481* | *0.615* |
| Adults (≥ 16 yrs) |  | **-6.7%** | **-10.8%** | **-13.1%** |  | **-12.1%** | **-9.0%** | **-8.8%** |
| *n* | *202* | *64* | *86* | *52* | *240* | *78* | *75* | *87* |
| *p-value* |  | *0.132* | *0817* | *0.173* |  | *0.080* | *0.394* | *0.361* |
| Severe genotypes |  | **-10.5%** | **-10.0%** | **-12.5%** |  | **-12.2%** | **14.4%** | **-9.9%** |
| *n* | *234* | *73* | *99* | *62* | *272* | *99* | *87* | *86* |
| *p-value* |  | *0.240* | *0.450* | *0.105* |  | *0.938* | *0.863* | *0.937* |
| Not on CFTR modulator treatment |  | ***-9.7*** | ***-10.3*** | ***-12.2*** |  | ***-12.4*** | ***-9.7*** | ***-10.5*** |
| *n* | *254* | *90* | *113* | *51* | *308* | *107* | *101* | *100* |
| *p-value* |  | *0.323* | *0.189* | ***0.037*** |  | *0.110* | *0.239* | *0.675* |
| On inhaled antibiotics |  | ***-10.0*** | ***-10.5*** | ***-14.4*** |  | ***-12.5*** | ***-10.9*** | ***-10.0*** |
| *n* | *237* | *88* | *106* | *43* | *281* | *100* | *89* | *92* |
| *p-value* |  | *0.576* | *0.269* | *0.576* |  | *0.623* | *0.792* | *0.601* |
| *p-value in 'Low' column compares Low vs Medium; p-value in 'Medium' column compares Medium vs High; p-value in 'High' column compares High vs Low, Percentage difference reflects change from 2008 to 2017 within each exposure category* | | | | | | | | |

### Supplementary Figures

#### **Supplementary Figure S1**: **Directed acyclic graph (DAG).** Associations between exposure, potential mediator and outcome in people with CF in the London, UK CF Registry. Exposures are shown in blue; outcomes in orange; mediators in green; effect modifiers in purple; covariates in white; and confounders in yellow. Grey boxes indicate variables not taken into account in our analysis.

#### **Supplementary Figure S2**: **Flow diagram.** Inclusion and exclusion criteria used for people with CF in the London, UK CF Registry

#### **Supplementary Figure S3**: Overall mean ppFEV_1_ trend among people with CF with a residential postcode in London between 2008 and 2017 (n=393).

**Supplementary Figure S4**: **Lung function and PM_2.5_ in CF “non-movers”**. Unadjusted (A) and adjusted (B) estimated change in mean ppFEV_1_ associated with annual exposure of PM_2.5_ among people with CF who did not change their residential postcode in London between 2008 and 2017 (n=110).

The adjusted model accounted for age at baseline, sex, height, weight, CFTR genotype, *P. aeruginosa* positive culture and deprivation (Townsend index). Number of people with CF: low = 35, medium = 43, high = 32

**A**

**B**

#### **Supplementary Figure S5**: ***Lung function and PM_2.5_ in CF children*.** Unadjusted (A) and adjusted (B) estimated change in mean ppFEV_1_ associated with annual exposure of PM_2.5_ among children with CF (6 – 15 years) with a residential postcode in London between 2008 and 2017 (n=96).

The adjusted model accounted for age at baseline, sex, height, weight, CFTR genotype, *P. aeruginosa* positive culture and deprivation (Townsend index). Number of children with CF: low = 27, medium = 40, high = 29

#### **Supplementary Figure S6**: **Lung function and PM_2.5_ in CF adults.** Unadjusted (A) and adjusted (B)estimated change in mean ppFEV_1_ associated with annual exposure of PM_2.5_ among adults with CF (≥16 years) with a residential postcode in London between 2008 and 2017 (n=202).

The adjusted model accounted for age at baseline, sex, height, weight, CFTR genotype, *P. aeruginosa* positive culture and deprivation (Townsend index). Number of adultswith CF: low = 64, medium = 86, high = 52

**A**

**B**

#### **Supplementary Figure S7 ‎**: **Lung function and PM_2.5_ in severe CF.** Unadjusted (A) and adjusted (B) estimated change in mean ppFEV_1_ associated with annual exposure of PM_2.5_ among people with CF with a severe genotype in London between 2008 and 2017 (n=234).

The adjusted model accounted for age at baseline, sex, height, weight, *P. aeruginosa* positive culture and deprivation (Townsend index). Number of people with CF: low = 73, medium = 99, high = 62

**A**

**B**

**Supplementary Figure S8‎**: **Lung function and PM_2.5_ in CF not on modulator.** Unadjusted (A) and adjusted (B) estimated change in mean ppFEV_1_ associated with annual exposure of PM_2.5_ among people with CF not on CFTR modulator treatment at any point between 2008 and 2017, in London *(n=254)*.

The adjusted model accounted for age at baseline, sex, height, weight, *P.* *aeruginosa positive culture* and deprivation (Townsend index). Number of people with CF: low = 90, medium = 113, high = 51

**A**

**B**

**Supplementary Figure S9‎**: **Lung function and PM_2.5_ in CF on inhaled antibiotics.** Unadjusted (A) and adjusted (B) estimated change in mean ppFEV_1_ associated with annual exposure of PM_2.5_ among people with CF who received inhaled antibiotic treatment between 2008 and 2017, in London *(n=237)*.

The adjusted model accounted for age at baseline, sex, height, weight, *P.* *aeruginosa positive culture* and deprivation (Townsend index). Number of people with CF: low = 88, medium = 106, high = 43

**A**

**B**

#### **Supplementary Figure S10:** **Lung function and NO_2_ in “CF non-movers”.** Unadjusted (A) and adjusted (B) estimated change in mean ppFEV_1_ associated with annual exposure of NO_2_ among people with CF who did not change their residential postcode between 2008 and 2017 (n=144).

The adjusted model accounted for age at baseline, sex, height, weight, CFTR genotype, *P. aeruginosa* positive culture and deprivation (Townsend index) Number of people with CF: low = 64, medium = 49, high = 31

**A**

**B**

**Supplementary Figure S11**: **Lung function and NO_2_ in CF children”.** Unadjusted (A) and adjusted (B)estimated change in mean ppFEV_1_ associated with annual exposure of NO_2_ among children with CF (6 – 15 years) with a residential postcode in London between 2008 and 2017 *(n=114)*.

The adjusted model accounted for age at baseline, sex, height, weight, genotype *P. aeruginosa* positive culture and deprivation (Townsend index). Number of children with CF: low = 50, medium = 41, high = 23

**A**

**B**

#### **Supplementary Figure S12**: **Lung function and NO_2_ in CF adults.** Unadjusted (A) and adjusted (B) estimated change in mean ppFEV_1_ associated with annual exposure of NO_2_ among adults with CF (≥16 years) with a residential postcode in London between 2008 and 2017 (n=240).

The adjusted model accounted for age at baseline, sex, height, weight, CFTR genotype, *P. aeruginosa* positive culture and deprivation (Townsend index). Number of adults with CF: low = 78, medium = 75, high = 87

**A**

**B**

**Supplementary Figure S13‎**: **Lung function and NO_2_ in severe CF.** Unadjusted (A) and adjusted (B) estimated change in mean ppFEV_1_ associated with annual exposure of NO_2_ among people with CF with a severe genotype in London between 2008 and 2017 *(n=272)*.

The adjusted model accounted for age at baseline, sex, height, weight, *P.* *aeruginosa positive culture* and deprivation (Townsend index). Number of people with CF: low = 99, medium = 87, high = 86

**A**

**B**

**Supplementary Figure S14‎**: **Lung function and NO_2_ in CF not on modulator.** Unadjusted (A) and adjusted (B) estimated change in mean ppFEV_1_ associated with annual exposure of NO_2_ among people with CF not on CFTR modulator treatment at any point between 2008 and 2017, in London *(n=308)*.

The adjusted model accounted for age at baseline, sex, height, weight, *P.* *aeruginosa positive culture* and deprivation (Townsend index). Number of people with CF: low = 107, medium = 101, high = 100

**A**

**B**

**Supplementary Figure S15‎**: **Lung function and NO_2_ in CF on inhaled antibiotics.** Unadjusted (A) and adjusted (B) estimated change in mean ppFEV_1_ associated with annual exposure of NO_2_ among people with CF who received antibiotic treatment between 2008 and 2017, in London *(n=281)*.

The adjusted model accounted for age at baseline, sex, height, weight, *P.* *aeruginosa positive culture* and deprivation (Townsend index). Number of people with CF: low = 100, medium = 89, high = 92

**A**

**B**

### Supplementary References

1.	ONS. *Office for National Statistics; Population and household estimates, England and Wales: Census 2021* [*https://www.ons.gov.uk/peoplepopulationandcommunity/populationandmigration/populationestimates/bulletins/populationandhouseholdestimatesenglandandwales/census2021*](https://www.ons.gov.uk/peoplepopulationandcommunity/populationandmigration/populationestimates/bulletins/populationandhouseholdestimatesenglandandwales/census2021) [Accessed 17th Jan 2024]. 2022;

2.	ONS. *Office for National Statistics; Postcode Headcounts and Household Estimates - 2011 Census.* <https://www.nomisweb.co.uk/census/2011/postcode_headcounts_and_household_estimates>. [Accessed 19th Aug. 2024]. 2013;

3.	Ordnance Survey. *Code-Point Open.* <https://www.ordnancesurvey.co.uk/products/code-point-open>. [Accessed 17th Jan. 2024]. 2024;

4.	ONS. *Office for National Statistics.. 2011 Census: Aggregate Data. [data collection]. UK Data Service. SN: 7427,* <http://doi.org/10.5257/census/aggregate-2011-2> [Accessed 19th Aug 2024]. 2020;

5.	R Core Team. R: A language and environment for statistical computing. R Foundation for Statistical Computing, Vienna, Austria. <https://www.R-project.org/>. 2022;
